# Effects of Electrical Muscle Stimulation on Waist Circumference in Adults with Abdominal Obesity: A Randomized, Double-blind, Sham-Controlled Trial

**DOI:** 10.31729/jnma.3826

**Published:** 2018-12-31

**Authors:** Eun Jung Choi, Yun Jun Kim, Sang Yeoup Lee

**Affiliations:** 1 Department of Family Medicine, Daedong Hospital, Busan, South Korea; 2 Department of Family Medicine, Pusan National University School of Medicine, Yangsan, South Korea; 3 Department of Medical Education, Pusan National University School of Medicine, Yangsan, South Korea; 4 Department of Family Medicine, Obesity, Nutrition and Metabolism Clinic and Research Institute of Convergence of Biomedical Science and Technology, Pusan National University Yangsan Hospital, Yangsan, South Korea

**Keywords:** *abdominal obesity*, *electric stimulation therapy*, *muscle*, *waist circumference*

## Abstract

**Introduction:**

We investigated the effects of electrical muscle stimulationon waist circumference as compared with an identical device providing transcutaneous electrical nerve stimulation as control in adults with abdominal obesity.

**Methods:**

This was a randomized, double-blind, sham-controlled trial. Sixty patients with abdominal obesity received electrical muscle stimulation or transcutaneous electrical nerve stimulation randomly five times a week for 12 weeks.

**Results:**

The electrical muscle stimulationgroup achieved a mean 5.2±2.8 cm decrease in waist circumference while the transcutaneous electrical nerve stimulation group showed only a 2.9±3.3 cm decrease (P=0.005). About 20 (70.0%) of the electrical muscle stimulation group lost more than 4 cm of waist circumference but that only 8 (33.3%) of the transcutaneous electrical nerve stimulation group did so (P=0.008). Furthermore, fasting free fasting acid levels were significantly higher in the electrical muscle stimulation than in the transcutaneous electrical nerve stimulationgroup at week 12 (P=0.006). In the electrical muscle stimulation group, slight decreases in visceral abdominal fat and total abdominal fat areas by computer tomography were observed at 12 weeks, but these decreases were not significant. In addition, patients' self-rated satisfaction scores with this program were significantly higher in the electrical muscle stimulation group.

**Conclusions:**

The 12-week electrical muscle stimulation program modestly reduced waist circumference in abdominally obese adults without side effects.

## INTRODUCTION

Obesity is one of the leading preventable causes of death worldwide and a major public health concern.^1,2^ Regional adiposity is more strongly correlated with cardiometabolic comorbidities than total body fat mass and in particular, that abdominal adiposity is an independent risk factor for coronary heart disease (CHD).^3^ Current obesity management places a focus on diet, physical exercise, life style modifications, medication, and surgery.^1^

However, electrical muscle stimulation (EMS) was recently introduced as a treatment tool for abdominal obesity.^4^ Initially, EMS was introduced as training program tool to improve muscular strength, but it is now used for muscle rehabilitation, treat obesity and improve body shape.^5,6^ EMS has been suggested to promote the formation of adenosine triphosphate,^7^ increase oxygen intake,^8^ decrease body fat by increasing muscle strength, improve blood circulation, accelerate waste product excretion though the lymphatic system,^5^ stimulate cell regeneration,^9^ and increase metabolic rate by increasing local temperature.^10^ Although a previous clinical trial reported reduced waist circumference (WC) after 8 weeks of EMS,^11^ unfortunately, no randomized controlled trial has evaluated the effect of EMS on obesity or regional fat distribution.

We hypothesized EMS offers an effective and safe treatment for men and women with abdominal obesity, and that EMS can decrease WC (abdominal fat), increase abdominal muscle mass, and improve biochemical markers. Therefore, we compared the efficacy and safety of 12 weeks of EMS treatment with those obtained using transcutaneous electrical nerve stimulation (TENS) as a sham control in adults with abdominal obesity.

## METHODS

This double-blind, randomized, sham-controlled trial was conducted at Pusan National University Hospital (PNUH) from January 2, 2014 to June 8, 2015. The study was approved by the Institutional Review Board at PNUH (No. 2003011)and the trial was registered with ClinicalTrials.gov (no. NCT02970812).

The study inclusion criteria included age between 18 and 65 years at screening and a WC of >90 cm for men or >80 cm for women.^12^ The exclusion criteria applied were: pregnancy, breastfeeding, taking medication for weight loss or any treatment including medication known to affect weight, a weight loss of >3% in the preceding 3 months, major surgery during the 1 year prior to study commencement, and the presence of any metal containing implant. Subjects with the following were also excluded; aspartate aminotransferase (AST) or alanine aminotransferase (ALT) greater than 2.5 fold the upper reference limit, serum creatinine (Cr) greater than the upper reference limit, a history of CHD, major organ dysfunction, cancer, a severe lung disease, severe cerebral trauma, uncontrolled hypertension, or a diagnosed psychiatric disease (including eating disorder). Seventy participants were initially screened to determine eligibility. Five participants met the exclusion criteria and five participants declined to participate. Finally, 60 (85.7%) participants were enrolled and then, participants were randomly assigned to one of two groups: the EMS group (n=30, group using a EMS device) or the control group (n=30, group using a TENS device, which externally was identical to the EMS device).

Participants were randomly assigned to two groups of equal number using random number tables and assigned identification numbers on recruitment. To minimise potential bias, participating participants were not informed of their study allocation until after they had provided consent. No investigator or member of other staff interacting with participants also was aware of study group assignments for the duration of the trial. Participants were treated with EMS or TENS using 66 min sessions, 5 days per week for 12 weeks by using a prototype device named TGBODY-4CH (serial number 612001 ~ 612030, 612061 ~ 612090) developed by NS-Medicom (Gimhae, South Korea) and approved by the Korea Testing Certification Medical Device Center (#2004-0042, KTC, Gunpo, South Korea). This device provided EMS and TENS programs, which were selected by pressing a button. Control group was treated by applying current regularly at a frequency of 1 Hz, which was determined basis on endorphin theory.^13^ The electric current applied during TENS can cause muscle movement, but this does not constitute an effective muscle contraction exercise.^14^ Pads were applied to the abdomen, rectus abdominis and external oblique abdominal muscle areas, so as to avoid any recent wound, infection, scar, or wart, in the supine position. EMS program consisted of a warm-up, contraction and relaxation, cool-down, and was set to resemble actual muscle action during voluntary exercise (Table 1).

**Table 1:** Sequences of electrical muscle simulation and transcutaneous electrical nerve stimulation.

Action sequence	Freq. (Hz)	Width µ /s)	Action time/ Resting time (sec)	Total duration (min) × (times) = (min)
EMS				
Warm-up	5	250	2/3	3 × 2 = 6
Contraction	55	300	10/10	10 × 2 = 20
Warm-up	6	180	2/3	5 × 2=10
Contraction	65	300	10/10	10 × 2=20
Warm-down	4	160	2/3	5 × 2=10
TENS				
Continuous	1	150	Continuous	66

*EMS, electrical muscle simulation; TENS, transcutaneous electrical nerve stimulation*

Alcohol consumption, smoking status, and history of comorbidity were collected using self-administered postal questionnaires. WC was tape-measured twice at the midpoint between the lower margin of the least palpable rib and the top of the iliac crest at the end of a normal expiration and recorded to 0.1 cm.^15^

Visceral, subcutaneous and total adipose tissue (VAT, SAT and TAF) areas (expressed as cm^2^) were determined by CT (Somatome Plus-4 CT, Siemens, Erlangen, Germany) using a single tomographic slice at the L4-L5 disc space. Hounsfield unit cut-off values between 190 and 30 were assigned for adipose tissue in CT images.

At baseline and after the 12-week treatment period, blood samples were taken after at least an 8-hour fast for general blood testing. Glucose was measured using a glucose oxidase test method (LX-20, Beckman Coulter, Fullerton, CA, USA). AST, ALT, Cr and lipid profile were determined using a Toshiba TBA200FR biochemical analyser (Toshiba Co. Ltd., Tokyo). Serum insulin was measured using a solid-phase 125I radioimmunoassay (Coat-A-Count® Insulin, Diagnostic Products Corp., Los Angeles, CA). A homeostasis model assessment indexinsulin resistance (HOMA-IR) of >2.34 was considered to indicate the presence of insulin resistance in relation to metabolic syndrome.^16,17^

The start of the study, subjects were asked to maintain their usual consumption and physical activity levels throughout the study period. Nutrition assessments were conducted at baseline and at 12 weeksusing a 24-hour recall interview and physical activity (MET-minutes) using the Short-Form International Physical Activity Questionnaire.^18^

Patients' self-rated satisfactions with treatment were measured weekly during the trial period using a scale ranging from zero to 5, which represented lowest and highest satisfaction levels, respectively.

The developments of adverse events were closely monitored, and participants were encouraged to report any potential adverse events throughout the study. Creatine kinase (CK) and lactate dehydrogenase (LDH) were measured using standard commercial kits to determine the presence of any possible muscle damage.

**Figure 1.**
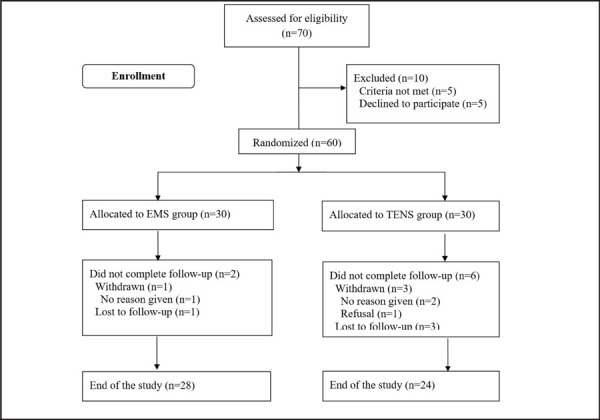
CONSORT Flowchart of participants' progress during the study.

A prior sample size calculations were based on the ability to detect a difference in mean WC of 4 cm after intervention using a standard deviation of 5 cm in WC obtained from a previous study.^11^ We estimated that 25 patients in each group would be required (two-tailed a = 0.05, ß = 0.20). To account for a potential dropout rate of 10%, 30 patients were recruited per group. Among 60 participants, two in the EMS group and six in the control group dropped out during the study without completing the study procedure (Figure 1).

The characteristics of these 8 participants were similar to those that completed the study. Accordingly, the analysis was conducted on 52 subjects (EMS group, n=28; control group, n=24). Categorical data are presented as frequency counts and percentages. The primary outcome variable was WC and the secondary outcome variables were patient satisfaction, VAT and SAT, and laboratory data at 12 weeks follow up. When the test data was unavailable, the last recorded data was used in the analysis (called the last observation carried forward). Efficacy analyses were based on the intent-to-treat (ITT) population of subjects who received at least EMS or TENS and had at least one assessment post-baseline. The within-group comparisons were done with a paired t-test or Wilcoxon signed-rank test when appropriate. We used linear mixed-effects modelling for repeated measures over time with WC, patient satisfaction, VAT, SAT, and laboratory data as the dependent variables and effects for time (baseline, 12 weeks), group (EMS, control), and time by group interaction as independent variables, with adjustment for baseline total calorie intake and physical activity as covariates. In addition, WC loss was categorized as follows: <2 cm, 2 cm to 4 cm, or >4 cm. Chi-square test was used to compare proportions with the WC loss categories before and after treatment. P values of less than 0.05 were considered statistically significant. We used SAS version 9.1 (SAS Institute, Cary, NC, USA) for all analyses.

## RESULTS

Of the total sample, 52 (74.3%) completed the 12-week follow-up for the primary endpoint. No significant intergroup differences were observed between general characteristics at baseline (Table 2).

**Table 2:** Baseline characteristics.

Variance	Control group (n = 30)	EMS group (n=30)	P ^*^
Age (years)	40.0±12.9	38.5±10.6	0.625
Male (%)	5 (16.7)	9 (30.0)	0.222
Diseases^†^	2 ( 6.7)	2 (6.7)	1.000
Medications (%)^‡^	2 ( 6.7)	2 (6.7)	1.000
Regular exercise (%)^§^	6 (20.0)	8 (26.7)	0.493
Current smoker (%)	2 ( 6.7)	4 (13.3)	0.389
Alcohol drinker (%)^∥^	9 (30.0)	11 (36.7)	0.584

*Results are expressed as means ± SD or numbers (%)*.

* *Two-sample t-test or chi-square test*

† *2 subjects were diagnosed with hypertension, 1 with atopic dermatitis and 1 with asthma.*

‡ *Hncluding antihypertensive drugs, antihistamine*

§ *At least 30minutes per day on five or moredays per week^32^*

∥ *≥2 drinks (28 g) for men and ≥1 drinks (14 g) for women per day, based on standard drink sizes described in US guidelines.^33^*

Four participants reported a diagnosis of hypertension, atopic dermatitis, or asthma. No intra- or intergroup group differences were found for caloric intake or physical activity throughout the 12-week trial (Table 3).

**Table 3:** Changes in anthropometric, metabolic, and biochemical characteristics between baseline and week 12 (ITT analysis set).

	Control group (n = 30)				EMS group (n = 30)				
	Week 0	Week 12	A 0–12 weeks	P^*^	Week 0	Week 12	A 0–12 weeks	P^*^	P^†^
**Anthropometric characteristics**									
Height (cm)	162.6 ± 7.6			NA	164.5±7.2			NA	NA
Weight (kg)	72.5±11.7	72.0±12.3	0.4±1.4	0.106	72.9±12.5	71.8±12.8	1.1 ± 1.6	0.001	1.000
BMI (kg/m^2^)	27.4 ±3.5	27.2±3.8	0.2±0.5	0.110	26.8±3.2	26.4±3.3	0.4 ± 0.6	0.001	0.091
WC (cm)	92.3±7.2	89.4±9.5	2.9±3.3	<0.001	92.2±11.6	87.0±12.4	5.2 ± 2.8	<0.001	0.005
WC (%)	100.0	96.7±3.8	3.3±3.8	<0.001	100.0	94.2±3.1	5.8 ± 3.1	<0.001	0.027
VAF (cm^2^)	105.7±66.3	105.3 ±65.3	0.3±20.8	0.928	91.0±48.3	85.2±47.2	5.7 ± 23.3	0.187	0.348
SAF (cm^2^)	217.8±63.7	211.1±69.3	6.7±26.7	0.181	210.0±76.5	205.5±84.4	4.4 ± 35.3	0.498	0.780
TAF (cm^2^)	323.4±82.1	316.4±91.2	7.0±28.5	0.186	300.9±103.4	290.7±111.7	10.2 ± 38.5	0.159	0.722
Metabolic and biochemical characteristics									
TC (mg/dL)	192.1±27.9	195.2±32.4	−3.1±20.0	0.397	183.5±33.2	179.8±30.0	3.7 ± 23.5	0.399	0.232
TG (mg/dL)	110.1±61.7	122.1±71.6	−12.0±43.9	0.147	122.5±62.5	115.9±51.6	6.5 ± 57.2	0.537	0.165
HDL-C (mg/dL)	54.9±12.7	56.3±11.2	−1.4±7.1	0.276	55.7±12.2	57.4±12.1	−1.7 ± 8.1	0.271	0.906
cLDL-C (mg/dL)	115.2±27.9	114.5±32.7	0.7±17.0	0.824	103.2±33.6	99.2±29.4	4.0 ± 17.7	0.224	0.460
Insulin (µIU/ ml)	8.4±5.6	11.4±10.7	−3.0±10.2	0.117	8.5±5.0	11.0±12.3	−2.5 ± 11.5	0.241	0.854
Glucose (mg/dL)	86.9 ±11.1	91.2±14.1	−4.3±16.9	0.175	90.9±12.6	91.9±15.4	−0.9 ± 15.6	0.745	0.426
HOMA-IR	1.9 ±1.4	2.7±3.2	−0.9±3.2	0.154	1.9±1.3	2.6±3.2	−0.7 ± 3.1	0.247	0.822
FFA (µmol/)	414.8 ±219.6	349.6±192.2	65.2±213.3	0.105	354.2±136.1	435.4±222.6	−81.2 ± 186.8	0.024	0.006
hsCRP (mg/dL)	0.19±0.42	0.16±0.23	0.0±0.3	0.598	0.11 ±0.16	0.22±0.61	−0.1 ± 0.6	0.399	0.322
Calorie intakes and physical activities									
Total calorie intake (kcal/ day)	1873.3±346.1	1866.4 ±374.5	6.9 ±416.8	0.928	1943.1±399.9	2022.0 ±582.2	−79.0 ± 418.2	0.310	0.429
Physical activity (MET-minutes/week)	862.5 (83.0∼4493.0)	1011.0 (300.0∼11208.0)	45.0 (−3263.0∼8118.0)	0.162	1402.5 (108.0∼6978.0)	1665.0 (345.0∼14076.0)	138.0 (−2274.0∼7098.0)	0.045	0.393

*EMS, electrical muscle simulation; ITT, intention to treat; BMI, body mass index; WC, waist circumference; CT, computerized tomography; TAF, total abdominal fat area; VAF, visceral abdominal fat area; SAF, subcutaneous abdominal fat area; TC, total cholesterol; TG, triglyceride; HDL-C, high density lipoprotein cholesterol, cLDL-C, calculated low density lipoprotein cholesterol; HOMA-IR, homeostasis model assessment insulin resistance index; FFA, free fatty acid; hsCRP, high sensitivity C-reactive protein; Δ 0–12 weeks, difference versus baseline.*

*Results are presented as Means ± SDs or medians and ranges.*

* *paired t-test (means ± SD) or Wilcoxon signed-rank test (medians and ranges) for intra-group comparisons*

† *Two-way repeated-measures ANOVA over time for inter-group comparisons, with adjustment for baseline total calorie intake and physical activity as covariates.*

### Primary outcome

**Figure 2.**
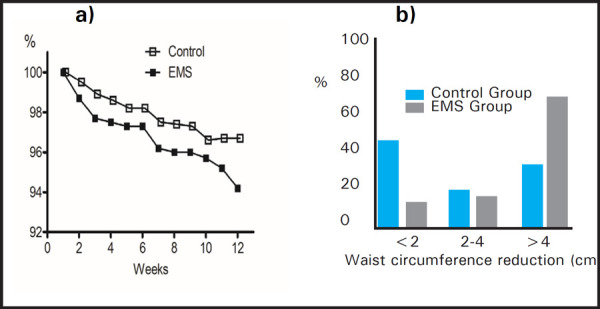
Effects of EMS on waist circumference. (a) Changes of waist circumference over the 12-week study period (ITT analysis set) EMS, electrical muscle simulation. P = 0.027 by repeated measures ANCOVA. ITT, intention to treat. (b) Intergroup comparison of waist circumference reductions achieved during the study period. P=0.008 by Chi-square test.

EMS group showed a 5.2±2.8 cm loss in WC after the trial period, whereas the control group showed a 2.9±3.3 cm loss, which was a significant difference (P=0.005, Table 3). In the EMS group, WC slightly declined more sharply during the latter half of the study (Figure 2a). Furthermore, participants were divided into subgroups according to degree of WC loss (<2 cm, 2 cm to 4 cm, and >4 cm), 20 (70.0%) of the EMS group were found to have lost >4 cm of WC, while only 8 (33.3% )of the control group did so (P = 0.008, Figure 2b).

### Secondary outcomes

The self-rated satisfaction scores were significantly greater in the EMS at study completion (3.9±0.7 vs. 2.8±1.2, P<0.001, data not shown). But no intergroup differences were found for abdominal fat distribution or other metabolic and biochemical characteristics throughout the trial period (Table 3). However, fasting FFA levels were significantly higher in the EMS group than in the control group at week 12 (P = 0.006, Table 3). Slight, non-significant, decreases in CT VAT and TAF areaswere observed in the EMS group over the 12 weeks.

### Safety

No adverse symptoms were encountered during the study, and CK, AST, ALT, and Cr did not change in either group (Table 4).

**Table 4:** Abnormal clinical findings at baseline and at study completion.

	Control group (n=30)		EMS group (n=30)		P^*^
	Week 0	Week 12	Week 0	Week 12	Week 0, 12
AST or ALT ≥1.5 × upper limit of normal	2 (6.7)	2 (6.7)	4 (13.3)	3 (10.0)	0.671, 1.000
Creatinine ≥1.5 mg/dl	0 (0.0)	0 (0.0)	0 (0.0)	0 (0.0)	NA, NA
CK≥1.5 × upper limit of normal	1 (3.3)	3 (10.0)	1 (3.3)	4 (13.3)	1.000, 0.353

*AST, aspartate transaminase; ALT, alanine transaminase; CK, creatine kinase*

*Results are expressed as frequencies (percentages).*

* Fisher's exact test within group

## DISCUSSION

The present study revealed that EMS is capable of producing physiological responses similar to those of cardiovascular exercise at mild to moderate intensities, despite the fact that EMS is performed without producing gross movement of limbs or loading joints. The EMS group achieved a modestly greater decrease in WC than the control group at week 12 (5.8% vs. 3.3%, P = 0.007). Furthermore, the proportion in the EMS group that achieved a WC reduction of >4cm was twice as high as that observed in the control group (Table 2). TENS only stimulates sensory nerves, whereas EMS excites motor nerves and results in the constriction skeletal muscle fibres, which means its metabolic benefits are similar to that of exercise. According to previous studies, 12 weeks of resistance exercise reduces WCs by 2–9% depending on initial Body Mass Index (BMI) and intensity of exercise.^19–22^ The contraction time of abdominal muscle exercise is about 3–5 seconds, but this contraction time can be tripled using EMS to increase energy consumption as high intensity exercise. In the present study, the time was set at 10 seconds as shown in Table 1. Repeating a 10–15 second-contraction time is difficult for laypeople without mechanical interventions. In the EMS group, WC declined more sharply in the latter half of the study (Figure 2a), presumably because EMS may have the potential to cause cumulative effects on WC. As skeletal muscle builds, energy is consumed more efficiently.^21^ To investigate the association between abdominal fat distributions with WC decreases, we measured visceral and subcutaneous fat areas by abdominal CT scan, but no intergroup differences were observed at week 12. Sharma et al. reported percentage body fat was found less after EMS,^5^ but this study was different from our research in the EMR program settings, research design, and research duration, measurement index, dietary or activity adjustment, using sham-control and so on. EMS does not appear to be comparable to cardiovascular aerobic exercise. There are several possible explanations for the discrepancy between WC and CT measures. First, the single CT slice taken at the L4-L5 disc space may not have matched WC level as defined by the WHO STEPS protocol.^15^ Second, the study period may have not been long enough to have produced a significant difference in abdominal visceral and subcutaneous fat areas. Muscle tension is also a critical confounder of WC as surrogate of abdominal obesity. Previous studies have shown that 3–5 months of combined aerobic plus resistance exercise was more effective at improving subcutaneous and visceral adiposity than aerobic only exercise in obese adolescents.^24,25^ However, WCs were not measured in either study. Nevertheless, other studies have demonstrated that regular aerobic exercise, with or without weight loss, is associated with reductions in total fat and/or visceral adipose tissue as determined by CT in obese individuals.^24,25^ Unfortunately, we did not combine aerobic exercise with EMS. Further research is required to compare EMS alone with EMS plus aerobic exercise.

In the present study, fasting FFA was significantly higher in the EMS group after the trial (P = 0.006, Table 3). Fasting FFA is a biomarker of lipolysis (the hydrolysis of triglycerides into glycerol and free fatty acid) in adipose tissue,^28,29^ although it can be considered as a risk factor for insulin resistance. Our finding suggests that EMS induces lipolysis in intramuscular adipose tissue, but on the other hand, other biomarkers were not altered by EMS. Furthermore, despite initial concerns, no significant abnormal clinical findings suggesting the presence of possible muscle damage were obtained and no participant complained of any adverse symptom (Table 4).

Some limitations of our study need to be mentioned. First, since this study was conducted over 12 weeks, no longer was performed. Second, in the study cohort the proportions of severely obese subjects (BMI>30 kg/m^2^, Asian-Pacific criteria), men and of those with a diagnosed disease were only 10.0%, 23.3%, and 6.7%, respectively, which means it may be difficult to apply our results to these populations. Furthermore, abdominal obesity was defined using the IDF Asia-Pacific criteria (women>80 cm). Third, no aerobic exercise or diet program was included in the present study, and thus, possible synergistic effect of EMS with aerobic exercise and/or diet were not explored. Furthermore, the majority of subjects recruited (75%) did not exercise regularly, and we would expect the effect of EMS to be greater in subjects that exercise regularly. Finally, despite significant declines in WC, there was much less reduction in abdominal adipose tissue mass and no improvement in metabolic outcomes. Despite all its limitations, to the best of our knowledge, the present clinical trial is the first randomized, controlled, double-blinded study to evaluate the effects of EMS on WC in adults.

## CONCLUSIONS

In summary, the 12-week EMS program was found to modestly reduce WC in abdominal obese adults without any evidence of side effects. These findings suggest EMS is an effective, safe auxiliary treatment for abdominal obesity in adults. A larger scale clinical trial is needed to confirm the results of this first-stage study and additional studies are required to assess the long-term effects of EMS on abdominal obesity.

## Conflict of Interest

**None.**

## References

[1] Ebbert JO, Elrashidi MY, Jensen MD. (2014). Managing overweight and obesity in adults to reduce cardiovascular disease risk. Curr Atheroscler Rep..

[2] Jensen MD, Ryan DH, Apovian CM, Ard JD, Comuzzie AG, Donato KA (2014). 2013 AHA/ACC/TOS guideline for the management of overweight and obesity in adults: a report of the American College of Cardiology/American Heart Association Task Force on Practice Guidelines and The Obesity Society. Circulation.

[3] Dhaliwal SS, Welborn TA, Goh LG, Howat PA. (2014). Obesity as assessed by body adiposity index and multivariable cardiovascular disease risk. PLoS One..

[4] Caulfield B, Crowe L, Coughlan G, Minogue C. (2011). Clinical application of neuromuscular electrical stimulation induced cardiovascular exercise. Conf Proc IEEE Eng Med Biol Soc..

[5] Sharma P, Lehri A, Verma SK. (2011). Effect of electrical muscle stimulation on reducing fat from the body. J Exer Sci Physiother.

[6] Porcari JP, McLean KP, Foster C, Kernozek T, Crenshaw B, Swenson C (2002). Effects of electrical muscle stimulation on body composition, muscle strength, and physical appearance. J Strength Cond Res..

[7] Valladares D, Almarza G, Contreras A, Pavez M, Buvinic S, Jaimovich E, Casas M. (2013). Electrical stimuli are anti-apoptotic in skeletal muscle via extracellular ATP. Alteration of this signal in Mdx mice is a likely cause of dystrophy. PLoS One..

[8] Minogue CM, Caulfield BM, Lowery MM. (2014). Whole body oxygen uptake and evoked torque during subtetanic isometric electrical stimulation of the quadriceps muscles in a single 30-minute session. Arch Phys Med Rehabil..

[9] Salmons S, Ashley Z, Sutherland H, Russold MF, Li F, Jarvis JC (2005). Functional electrical stimulation of denervated muscles: basic issues. Artif Organs..

[10] Watanabe K, Taniguchi Y, Moritani T. (2014). Metabolic and cardiovascular responses during voluntary pedaling exercise with electrical muscle stimulation. Eur J Appl Physiol..

[11] Porcari JP, Miller J, Cornwell K, Foster C, Gibson M, McLean K, Kernozek T. (2005). The effects of neuromuscular electrical stimulation training on abdominal strength, endurance, and selected anthropometric measures. J Sports Sci Med..

[12] Yoon YS, Lee ES, Park C, Lee S, Oh SW. (2007). The new definition of metabolic syndrome by the international diabetes federation is less likely to identify metabolically abnormal but non-obese individuals than the definition by the revised national cholesterol education program: the Korea NHANES study. Int J Obes (Lond)..

[13] Vance CG, Dailey DL, Rakel BA, Sluka KA. (2014). Using TENS for pain control: the state of the evidence. Pain Manag..

[14] Hartsell HD. (1986). Electrical muscle stimulation and isometric exercise effects on selected quadriceps parameters. J Orthop Sports Phys Ther..

[15] Shetty P. (2008). Waist circumference and waist-hip ratio.

[16] Wallace TM, Levy JC, Matthews DR. (2004). Use and Abuse of HOMA Modeling Diabetes Care.

[17] Lee S, Choi S, Kim HJ, Chung YS, Lee KW, Lee HC, Huh KB, Kim DJ. (2006). Cutoff values of surrogate measures of insulin resistance for metabolic syndrome in Korean non-diabetic adults. Korean Med Sci..

[18] Chun MY. (2012). Validity and reliability of Korean version of international physical activity questionnaire short form in the elderly. Korean J Fam Med..

[19] Herring LY, Wagstaff C, Scott A. (2014). The efficacy of 12 weeks supervised exercise in obesity management. Clin Obes..

[20] Kordi R, Dehghani S, Noormohammadpour P, Rostami M4, Mansournia MA. (2015). Effect of abdominal resistance exercise on abdominal subcutaneous fat of obese women: a randomized controlled trial using ultrasound imaging assessments. J Manipulative Physiol Ther..

[21] Skrypnik D, Bogdahski P, Madry E, Karolkiewicz J, Ratajczak M (2015). Effects of endurance and endurance strength training on body composition and physical capacity in women with abdominal obesity. Obese Facts..

[22] Choo J, Lee J, Cho JH, Burke LE, Sekikawa A, Jae SY. (2014). Effects of weight management by exercise modes on markers of subclinical atherosclerosis and cardiometabolic profile among women with abdominal obesity: a randomized controlled trial. BMC Cardiovasc Disord..

[23] Heinonen I, Kalliokoski KK, Hannukainen JC, Duncker DJ, Nuutila P, Knuuti J. (2014). Organ-specific physiological responses to acute physical exercise and long-term training in humans. Physiology..

[24] Alberga AS, Prud'homme D, Kenny GP, Goldfield GS, Hadjiyannakis S, Gougeon R, Phillips P, Malcolm J, Wells G, Doucette S, Ma J, Sigal RJ. (2015). Effects of aerobic and resistance training on abdominal fat, apolipoproteins and high-sensitivity C-reactive protein in adolescents with obesity: the HEARTY randomized clinical trial. Int J Obes..

[25] Dâmaso AR, da Silveira Campos RM, Caranti DA, de Piano A, Fisberg M, Foschini D, de Lima Sanches P, Tock L, Lederman HM, Tufik S, de Mello MT. (2014). Aerobic plus resistance training was more effective in improving the visceral adiposity, metabolic profile and inflammatory markers than aerobic training in obese adolescents. J Sports Sci..

[26] Lee S, Deldin AR, White D, Kim Y, Libman I, Rivera-Vega M, Kuk JL, Sandoval S, Boesch C, Arslanian S. (2013). Aerobic exercise but not resistance exercise reduces intrahepatic lipid content and visceral fat and improves insulin sensitivity in obese adolescent girls: a randomized controlled trial. Am J Physiol Endocrinol Metab.

[27] Lee S, Kuk JL, Davidson LE, Hudson R, Kilpatrick K, Graham TE, Ross R. (1985). Exercise without weight loss is an effective strategy for obesity reduction in obese individuals with and without Type 2 diabetes. J Appl Physiol.

[28] Stich V, de Glisezinski I, Berlan M, Bulow J, Galitzky J, Harant I, Suljkovicova H, Lafontan M, Rivière D, Crampes F. (1985). Adipose tissue lipolysis is increased during a repeated about of aerobic exercise. J Appl Physiol.

[29] Carmen GY, Victor SM. (2006). Signalling mechanisms regulating lipolysis. Cell Signal..

